# 959. Online CME Successful at Improving Knowledge, Competence and Confidence on Incorporating mAbs for COVID-19 Among a Global Audience

**DOI:** 10.1093/ofid/ofab466.1154

**Published:** 2021-12-04

**Authors:** Allison Armagan, Elaine Bell, Maria B Uravich, Shanthi Voorn

**Affiliations:** 1 Medscape, New York, New York; 2 Medscape Global, London, England, United Kingdom; 3 Medscape Education, New York, New York

## Abstract

**Background:**

The incorporation of effective treatments is critical to improving patient care for COVID-19. We assessed the educational impact of a series of continuing medical education (CME) activities on knowledge, competence, and confidence changes in US and OUS physicians related to the use of monoclonal antibodies (mAbs) for COVID-19.

**Methods:**

10 online, CME-certified activities were delivered in multiple formats. For individual activities, educational effect was assessed with a repeated pairs pre-/post-assessment study including a 1 to 7-item, multiple choice, knowledge/competence questionnaire and one confidence assessment question. To assess changes in knowledge, competence, and confidence, data were aggregated across activities and stratified by learning theme. McNemar’s test or paired samples t-test (P< .05) assessed educational effect. The activities launched between November 2020 and May 2021; data were collected through May 2021.

**Results:**

To date, the 10 activities have reached over 50,000 clinicians, including 24,627 physicians. Selected improvement/reinforcement in knowledge/competence measured as relative % change in correct responses pre/post education across the learning themes are reported. (i) 89% improvement/reinforcement among US ID specialists in knowledge/competence incorporating mAbs into patient care and 83% improvement among outside the US (OUS) ID specialists (*P* < .001). (ii) 70% improvement/reinforcement among US PCPs in knowledge/competence incorporating mAbs into patient care and 55% improvement among OUS PCPs (*P* < .001). (iii) 52% improvement/reinforcement in knowledge/competence among US PCPs regarding clinical data for mAbs and 44% among OUS PCPs (*P* < .001). (iv) 42% of US ID specialists and 29% of OUS ID specialists had a measurable improvement in confidence in identifying patients who would benefit from mAbs (*P* < .001).

**Conclusion:**

This series of online, CME-certified educational activities resulted in significant improvements in knowledge, competence, and confidence regarding the appropriate use of mAbs for SARS-CoV-2 in clinical practice. These results demonstrate the effectiveness of global curriculum-based education for clinicians designed to address specific gaps in care.

**Disclosures:**

**All Authors**: No reported disclosures

